# Emerging Roles and Potential Biological Value of CircRNA in Osteosarcoma

**DOI:** 10.3389/fonc.2020.552236

**Published:** 2020-10-28

**Authors:** Jiamei Liu, Liyu Yang, Qin Fu, Shengye Liu

**Affiliations:** ^1^Department of Pathology, The Shengjing Hospital of China Medical University, Shenyang, China; ^2^Department of Orthopedics, The Shengjing Hospital of China Medical University, Shenyang, China

**Keywords:** circular RNAs, osteosarcoma, prognosis, metastasis, epithelial mesenchymal transition

## Abstract

Circular RNAs (circRNAs) are endogenous noncoding RNAs that are widely found in eukaryotic cells. They have been found to play a vital biological role in the development of human diseases. At present, circRNAs have been involved in the pathogenesis, diagnosis, and targeted treatment of multiple tumors. This article reviews the research progress of circRNAs in osteosarcoma (OSA) in recent years. The potential connection between circRNAs and OSA cell proliferation, apoptosis, metastasis, and chemotherapy sensitivity or resistance, as well as clinical values, is described in this review. Their categories and functions are generally summarized to facilitate a better understanding of OSA pathogenesis, and findings suggest novel circRNA-based methods may be used to investigate OSA and provide an outlook for viable biomarkers and therapeutic targets.

## Introduction

Osteosarcoma (OSA) is the most common primary malignant bone tumor, accounting for 20% of all bone tumors and >5% of pediatric malignant tumors, with the highest incidence among children, adolescents, and the elderly (>50 years old) (1). The 5-year survival rate for nonmetastatic OSAs is 50–70%, but for metastatic OSAs (most commonly in the lungs), it is only 15–30% (2). So far, the pathogenesis and development of OSA remain unclear, and OSA treatment is still dominated by surgery and chemotherapy. For various reasons, outcomes for patients with OSA have not improved significantly in recent years mainly due to resistance of OSA cells to chemotherapy drugs (3). Therefore, further study into OSA pathogenesis is urgently needed, alongside the development of new and effective treatment regimens.

**Figure 1:**
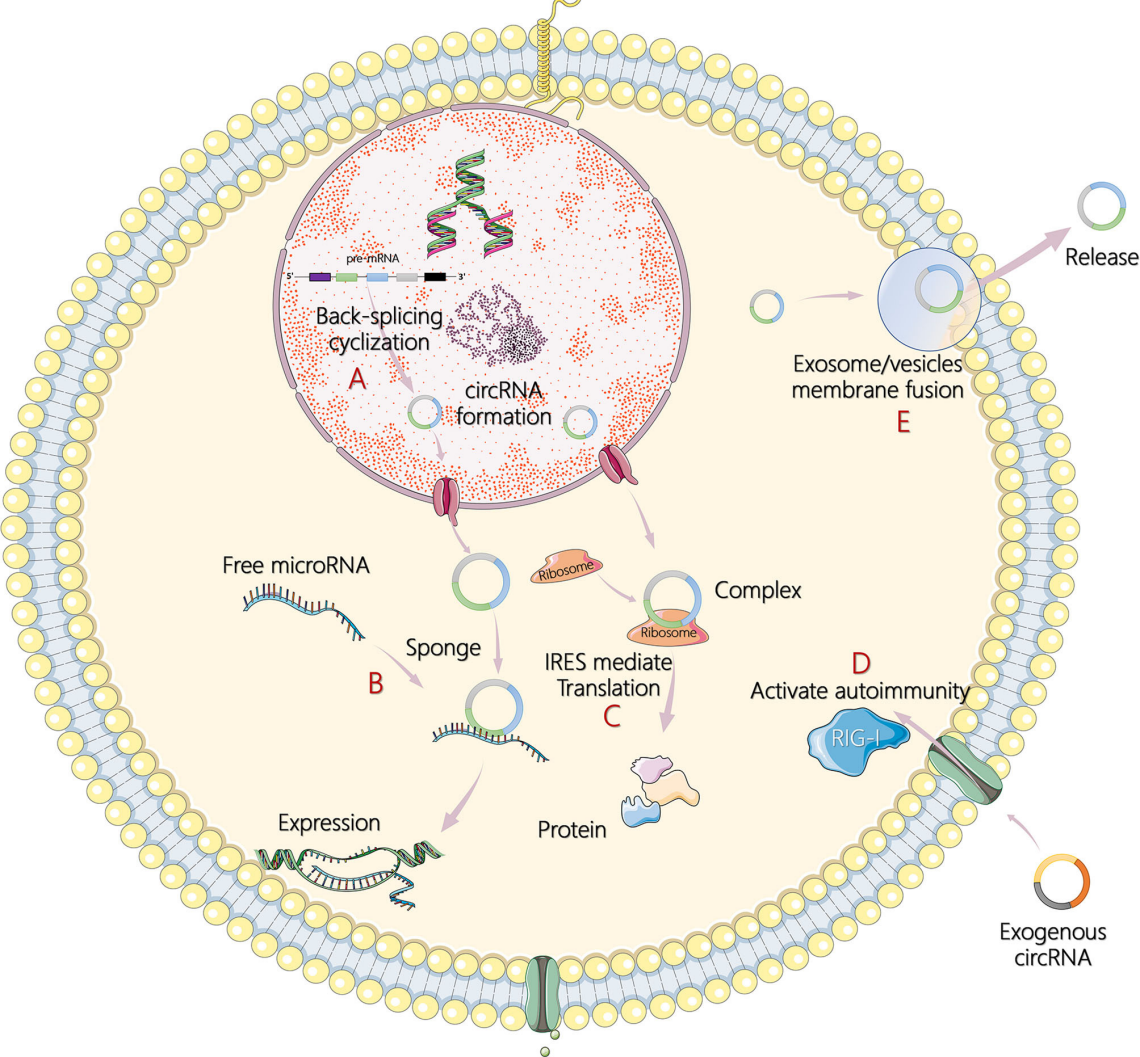
The main mechanisms of circular RNA (circRNA) in tumor pathogenesis. **(A)** Formation process of circRNA by back-splicing cyclization in cells. **(B)** CircRNAs act as sponges of microRNAs (miRNAs) and subsequently affect target gene expression. **(C)** CircRNAs encode itself by translation of proteins in an internal ribosome entry site (IRES)-mediated way. **(D)** Recognition of exogenous circRNAs activates retinoic acid inducible gene I (RIG-I)-mediated autoimmunity. **(E)** CircRNAs are transported outside cells to regulate responses through exosomes or extracellular vesicles by membrane fusion and release.

Less than 2% of the human genome's nucleic acid sequences encode proteins, and most genes are transcribed into noncoding RNA (ncRNA) (4). In recent years, circular RNA (circRNA) has become another research hit of ncRNA, following microRNA (miRNA) and long noncoding RNA (lncRNA). In 1976, Sanger et al. (5) and Kolakofsky (6) successfully discovered the existence of circRNA in plant viroids and sendai viruses. Electron microscopy was used in 1979 to clearly observe the circular structure of the circRNA in the cytoplasm of eukaryotic cells (7). However, at that time, circRNAs were regarded as abnormal RNA formed by the incorrect splicing of exon transcripts and, therefore, did not attract attention. In the 1990s, Nigro et al. (8) revealed that eukaryote protein-coding genes could form mature linear mRNA molecules. In addition, they discovered a special kind of reverse splicing reaction (back-splicing), which makes the exons sequence upstream and downstream (exon reverse cyclization), which eventually form a single closed loop structure with covalent bond connection (8). Rapid advances in RNA sequencing and bioinformatics have made large-scale analysis of transcriptome data a reality, and circRNAs have been found in a wide range of eukaryotes including humans, zebrafish, and fruit flies. Without A 3' end, 5' end, and poly A tail structure, circRNAs can escape the shear action of nucleic acid exonuclease such as RNase R, which is evolutionary conservative and stable than linear RNA, and more than 400 circRNAs can be detected even in human saliva (9–11). In addition, circRNAs also show cell specificity, tissue specificity, and sequence specificity (12). Studies on the biological functions of circRNAs are still at the exploratory stage. It has been found that circRNAs can act as competing endogenous RNAs, namely, miRNA sponges, to regulate the expression of target genes and can also act as transcription regulators or RNA-binding proteins to indirectly regulate genes at the posttranscriptional level (13, 14). Under certain conditions, circRNAs can even translate proteins directly (15). The main mechanisms of circRNA in tumor pathogenesis are illustrated in Figure 1.

Recently, researchers have made many new advances in multidisciplinary fields by using second-generation sequencing technology and gene chip analysis to screen differential circRNAs and validate their biological functions. It is now recognized that modulation of circRNA levels can result in a variety of molecular and physiological phenotype changes in cells, including effects on miRNAs, innate immunity, and many disease-relevant pathways (16). However, their characteristics in OSA remain far less documented. In this review, we will discuss the cellular mechanisms of the circRNAs in OSA tumorigenesis and progression.

## CircRNA and Cancer

CircRNAs have been studied in a variety of human tumor types, including those affecting the reproductive and urinary systems, digestive system, nervous system, respiratory system, etc. (17–21). Currently, the enormous potential for circRNA use in targeted therapy and as a noninvasive biomarker has garnered much attention from the broader scientific community (22). Numerous studies have shown that some circRNAs are involved in the pathogenesis of cancer and can be regarded as disease biomarkers or therapeutic targets (23). Noteworthy studies concerning the function of circRNAs in OSA pathogenesis and drug resistance have successively been reported, suggesting that they may provide valuable biomarkers for diagnosis or prognosis and show promise for the development of novel therapeutic approaches.

## CircRNA and Osteosarcoma

To investigate the differentially expressed circRNAs in OSA, researchers usually conduct a circRNA microarray screening, an analysis based on OSA/paired adjacent normal tissue or OSA/normal osteoblast cell lines. These approaches allow the identification of novel circRNAs that may be involved in the biological process of OSA progression. CircRNA-targeted miRNAs and miRNA-targeted genes can also be screened by microarray or bioinformatics analysis performed by software such as circRNA Interactome, miRDB, and Targetscan, etc. (24–26). CircRNAs could act as miRNA sponges to compete with endogenous RNAs in regulating posttranscriptional levels of gene expression (27). Functional bioinformatics analysis was selected for further research followed by experimental validation (28).

In this review, we summarize the roles of circRNAs in the proliferation, apoptosis, metastasis, and chemotherapy sensitivity or resistance, as well as the prognosis of OSA. We also summarized the emerging OSA–circRNAs identified and collate circRNA symbols corresponding to circRNA ID in the circBase database. In addition, the possible mechanisms of action of circRNAs are characterized.

## Regulation Mechanism of CircRNA on Osteosarcoma

Most aberrantly expressed circRNAs may serve as crucial regulators of OSA progression through the modulation of multifarious cancer hallmarks, functioning to deregulate cellular energetics, sustain proliferative signaling, promote invasion and metastasis, induce angiogenesis, and promote tumor immunology (29). Upregulated and downregulated circRNAs as well as their various mechanisms in OSA are represented in Tables 1, 2, respectively.

**Table 1:** Characterization of upregulated circular RNAs (circRNAs) as potential diagnostic biomarkers of osteosarcoma (OSA).

	**CircRNA**	**Expression**	**Gene symbol**	**Role of circRNA**	**Sponge microRNA/Target gene/Intersection molecules**	**References**
1	circITCH	Up	ITCH	Proliferation (+), Migration (+), Invasion (+)	Sponge miR-7	(30)
2	Hsa_circ_0000006 (Alias:circ_001621)	Up	SLC35E2B	Proliferation (+), Migration (+), Metastasis (+) Overall survival (−)	Sponge miR-578 (+) VEGF (+) CDK4 (+) MMP9	(31)
3	Hsa_circ_0000285	Up	HIPK3	Proliferation (+), Migration (+), Metastasis (+)	Sponge miRNA-599 (+) TGFB2	(32)
4	Hsa_circ_0001658	Up	ARID1B	Apoptosis (−), Proliferation (+), Migration (+), Invasion (+), Metastasis (+)	Sponge miR-382-5p (+) YB-1	(33)
5	Hsa_circ_0001162	Up	MMP9	Apoptosis (−), Proliferation (+), Migration (+), Invasion (+) Overall survival (−) TNM stage (+)	Sponge miR-1265 (+) CHI3L1	(34)
6	Hsa_circ_0102049	Up	ATL1	Apoptosis (−), Proliferation (+), Migration (+), Invasion (+), Tumor size (+), Pulmonary metastasis (+), Overall survival (−)	Sponge miR-1304-5p (+) MDM2	(35)
7	Hsa_circ_0000479	Up	EPSTI1	Proliferation (+), Migration (+)	Sponge miR-892b (+) MCL1	(36)
8	Hsa_circ_0009112	Up	ANKIB1	Proliferation (+), Invasion (+)	Absorb/stabilize miR-19b (−) SOCS3 (+) STAT3	(37)
9	Hsa_circ_0071989	Up	MYO10	Proliferation (+), EMT (+)	Sponge miR-370-3 (+) RUVBL1	(38)
10	circLRP6	Up	LRP6	Apoptosis (−), Proliferation (+), Migration (+), Invasion (+)	(−) KLF2 (−) APC	(39)
11	Hsa_circ_0004846	Up	SAMD4A	Proliferation (+), OSA stemness (+)	Sponge miR-1244 (+) MDM2	(40)
12	Hsa_circ_0001785	Up	ELP3	Apoptosis (−), Proliferation (+)	Sponge miR-1200 (+) HOXB2	(41)
13	Hsa_circ_0006101	Up	ORC2	Apoptosis (−), Proliferation (+), Invasion (+)	Absorb/stabilize miR-19a (−) PTEN (+) Akt	(42)
14	Hsa_circ_0023404 (circRNA_100876)	Up	RNF121	Apoptosis (−), Proliferation (+), Migration (+) Overall survival (−)	Sponge miR-136	(43)
15	circTADA2A	Up	TADA2A	Proliferation (+), Migration (+), Invasion (+), Tumorigenesis (+), Metastasis (+)	Sponge miR-203a-3p (+) CREB3	(44)
16	Hsa_circ_0000885	Up	INSR	Disease-free survival/Overall survival (−)		(45)
17	Hsa_circ_0000502	Up	None	Apoptosis (−), Proliferation (+), Migration (+), Invasion (+)	Sponge miR-1238	(46)
18	circFAT1	Up	FAT1	Proliferation (+), Migration (+), Invasion (+), Tumorigenesis (+)	Sponge miR-375 (+) YAP1	(47)
19	Hsa_circ_0001946 (Alias:CDR1as)	Up	CDR1	Apoptosis (−), Proliferation (+), Migration (+), EMT (+)	Sponge miR-7 (+) EGFR, (+) CCNE1, (+) PI3KCD, (+) RAF1, (+) N-cadherin, (−) E-cadherin, (+) PCNA	(48, 49)
20	Hsa_circ_0001721	Up	CDK14	Apoptosis (−), Proliferation (+), Migration (+), Invasion (+)	Sponge miR-569 Sponge miR-599	(50)
21	Hsa_circ_0000677 (Alias:circ_001569)	Up	ABCC1	Proliferation (+) Chemotherapy resistance (+)		(51)
22	Hsa_circ_0092509	Up	NT5C2	Apoptosis (−), Proliferation (+), Invasion (+), Tumor growth (+) Disease-free survival/Overall survival (−)	Sponge miR-448	(52, 53)
23	Hsa_circ_0081001	Up	CYP51A1	Overall survival (−)		(54)
24	Hsa_circ_0003998	Up	ARFGEF2	Proliferation (+), Invasion (+)	Sponge miR-197-3p (+) KLF10	(55)
25	Hsa_circ_0004674	Up	ADAM22	Overall survival (−)	Sponge miR-490-3p-ABCC2 Sponge miR-1254–EGFR	(28)
26	Hsa_circ_0032462 Hsa_circ_0028173 Hsa_circ_0005909	Up Up Up	SIPA1L1 ATP2A2 XPR1		Sponge miR-338-3p Sponge miR-142-5p (+) CADM1 (Potential target by bioinformatics)	(56)
27	Hsa_circ_0007534	Up	DDX42	Apoptosis (−), Proliferation (+) Tumor size (+) Overall survival (−)		(57)
28	Hsa_circ_0092340	Up	NASP	Proliferation (+), Invasion (+)	Sponge miR-1253 (+) FOXF1	(58)
29	Hsa_circ_0008717	Up	ABCB10	Apoptosis (−), Proliferation (+), Migration (+), Invasion (+)	Sponge miR-203 (+) Bmi-1	(59)
30	Hsa_circ_0051079	Up	AKT2	Proliferation (+), Migration (+), Invasion (+) Overall survival (−)	Sponge miR-26a-5p (+) TGF-β1	(60)
31	Hsa_circ_0001821	Up	PVT1	Proliferation (+), Chemotherapy resistance (+) Overall survival (−) Invasion (+), Metastasis (+), EMT (+)	(+) ABCB1 Sponge miR-205-5p (−) E-cadherin, (+) N-cadherin, (+) Vimentin, (+) c-FLIP	(61, 62)
32	Hsa_circ_0001564	Up	CANX	Apoptosis (−), Proliferation (+)	Sponge miR-29c-3p	(63)
33	Hsa_circ_0009910	Up	MFN2	Apoptosis (−), Proliferation (+)	Sponge miR-449a (+) IL6R	(64)
34	circUBAP2	Up	UBAP2	Apoptosis (−), Proliferation (+) Tumor size (+) Overall survival (−)	Sponge miR-143 (+) Bcl-2	(65)
35	circRNA_103801	Up	—		miR-370-3p/hsa-miR-338-3p/ miR-877-3p (Potential target by bioinformatics)	(66)
36	Hsa_circ_0056288	Up	GLI2	Proliferation (+), Migration (+), Invasion (+)	Sponge miR-125b-5p	(67)
37	Hsa_circ_0016347	Up	KCNH1	Proliferation (+), Migration (+), Invasion (+), Pulmonary metastasis (+)	Sponge miR-214 (+) Caspase-1	(68)
38	Hsa_circ_0041103	Up	TCF25	Viability (+), Proliferation (+), Migration (+), Invasion	Sponge miR-206 (+) MEK/ERK, (+) AKT/mTOR	(69)

**Table 2:** Characterization of downregulated circular RNAs (circRNAs) as potential diagnostic biomarkers of osteosarcoma (OSA).

	**CircRNA**	**Expression**	**Gene symbol**	**Role of circRNA**	**Sponge microRNA/Target gene/Intersection molecules**	**References**
1	circLARP4	Down	LARP4	Chemosensitivity (+) Disease-free survival/Overall survival (+) Enneking stage (−)	Sponge miR-424	(70)
2	Hsa_circ_0001258	Down	PPP6R2		Sponge miR-744-3p (+) GSTM2	(71)
3	Hsa_circ_0002052	Down	PAPPA	Apoptosis (+), Proliferation (−), Migration (−), Invasion (−)	Sponge miR-1205 (+) APC2	(72)
4	Hsa_circ_0000284	Down	HIPK3	Proliferation (−), Migration (−), Invasion (−) Lung metastasis and poor prognosis (−) Enneking stage (−)		(73)
5	circRNA_104980	Down			miR-1298-3p/miR-660-3p (Potential target by bioinformatics)	(66)
6	circITCH	Down	ITCH	Apoptosis (+), Viability (−), Proliferation (−), Migration (−), Invasion (−)	Sponge miR-22	(74)
7	Hsa_circ0021347	Down	SOX6	Enneking stage (−) Overall survival (+)	Sponge B7-H3	(75)
8	Hsa_circ_0000190	Down	CNIH4	Proliferation (−), Migration (−), Invasion (−)	Sponge miR-767-5p (−) TET1	(76)

## Onco-CircRNA (Upregulated CircRNA)

At present, most circRNA studies conducted are based on elevated circRNAs in OSA. Oncogenic circRNAs can participate in inducing the progression of OSA. Their oncogenic function involves promotion of cell proliferation, colony formation, migration, and invasion, as well as affecting the rate of apoptosis. Some circRNAs have also been identified as closely correlated with OSA prognosis. For example, compared with adjacent tissues, circ_0001658 displayed a significantly higher expression in OSA tissues. Hsa_circ_0001658 could impede apoptosis by sponging miR-382-5p and positively modulating Y-box binding protein 1 (YB-1) expression to facilitate the proliferation, migration, and invasion of OSA cells (33). Wu et al. (44) revealed that increased circTADA2A expression in OSA tissue and cells promotes the progression and metastasis of OSA by sponging miR-203a-3p and by targeting oncogene cyclic AMP-responsive element-binding protein 3 (CREB3), both functionally and mechanistically. CircRNAs shsa_circ_0032462, hsa_circ_0005909, and hsa_circ_0028173 were found to be overexpressed in human OSA and to promote cell adhesion molecule 1 (CADM1) expression by functioning as miRNA sponges (56). Similar regulatory mechanisms were found in other studies, such as for circCANX (63), hsa_circ_0009910 (64), and hsa_circ_0056288 (67).

Of note, a study conducted by Du et al. (37) found that circANKIB1 could play an absorbing role with miR-19b, and that both molecules were upregulated in OSA cells (77). This study further found that circANKIB1 promoted miR-19b expression through absorption, thereby inhibiting the expression of *SOCS3*, a downstream target gene, and activating the signal transducer and activator of transcription 3 (STAT3) pathway to promote OSA progression. Another similar study indicated that circORC2 could adsorb miR-19a to stabilize its inhibitory function on target gene phosphatase and tensin homolog (PTEN) expression and activate downstream Akt pathway (42). Currently, most studies have shown that circRNAs exert effects on target genes by competitive binding to miRNAs (78). However, circRNAs stabilize miRNA functions through adsorption and enhance regulation of target genes by reducing the degradation of miRNAs (79), which may represent a novel mechanism. These studies have provided a new research method based on circRNA–miRNA–target gene axis and demonstrate the potential of circRNAs as OSA-targeted therapies.

The Enneking surgical staging system has been used for classification of musculoskeletal tumors by surgeons around the world. It is characterized by reliability, reproducibility, and prognostic importance for musculoskeletal sarcomas, especially for those originating in the axial skeleton. Some circRNAs well reflected the stage of OSA, which indicated its clinicopathological features (80). Hsa_circ_0000885 expression was significantly increased in tissue and serum samples from patients with OSA compared with controls, and expression levels increased with Enneking stage IIB and III OSA compared with early-stage OSA. Receiver operating characteristic (ROC) curve analysis suggested that hsa_circ_0000885 may act as a good diagnostic biomarker for OSA (45).

## Tumor-Suppressor CircRNA (Downregulated CircRNA)

CircRNAs can also act as tumor suppressants to inhibit tumor growth. The study of tumor-suppressor circRNA will provide a new direction for the diagnosis, treatment, and prognosis of OSA. In OSA, circRNA can act as a miRNA sponge to indirectly downregulate the expression of target genes, thus playing a role in cancer inhibition. For instance, underexpressed circRNA hsa_circ_0002052 was screened out and validated in OSA tissues and in OSA cells, which might be a potential therapeutic target for OSA intervention. Hsa_circ_0002052 suppressed Wnt/β-catenin activation by promoting APC2 expression through sponging miR-1205, which led to delayed OSA progression (72). McEachron et al. (81) and Wang et al. (82) concluded that hsa_circ0021347 was selected and validated to be significantly downregulated in OSA tissues, and cell lines showed a strong negative relationship with B7-H3, which served as a negative regulator of osteoimmunology, helping tumor cells escape immune surveillance. Furthermore, the hsa_circ0021347–miR-646-NOB1 axis was suggested to be involved in promoting tumor differentiation and invasion in OSA; however, this needs to be validated in future studies (75).

Research has found hsa_circ_0000190 to exhibit an obvious reduction in extracellular nanovesicles (EVs) and tissues of OSA patients. Most hsa_circ_0000190 was discovered to be encapsulated in EVs. EVs containing hsa_circ_0000190 in OSA cells transported from normal cells could block biological malignant behaviors by inhibiting the migration, proliferation, and invasion both *in vitro* and *in vivo*. In addition, EVs containing hsa_circ_0000190 might induce miR-767-5p to modulate TET1 and impede OSA progression. Li et al. (76) also offer a new concept for circRNA therapy based on cell–cell communication by packaging into circRNAs. Several other downregulated circRNAs, listed in Table 2, will be analyzed in details in the subsequent sections.

## CircRNA and Osteosarcoma Metastasis

The metastasis of OSA depends on many pathological processes and regulation of cytokines. Epithelial-mesenchymal transition (EMT) refers to the transformation of epithelial cells into cells with mesenchymal phenotype. The main characteristic of EMT is that epithelial cells lose their original polarity but gain mesenchymal characteristics. The EMT biological process plays an important role in tumor metastasis, with tumor cells that lose the bond among cells being much more able to invade and metastasize (83). Chen et al. (38) showed that circMYO10 regulated EMT and activated Wnt/β-catenin signaling, thereby regulating the miR-370-3p/RUVBL1 axis to promote H4K16Ac at the promoter region of β-catenin/LEF1 target genes. It was also found that cerebellar degeneration-related protein 1 (CDR1) knockdown led to the inhibition of transforming growth factor-β (TGF-β)-induced EMT by upregulating the mesenchymal phenotype with increased N-cadherin and downregulated the epithelial phenotype with reduced E-cadherin (48, 49). EMT occurrence and development are always accompanied by multiple molecular interactions and signaling pathways, including TGF-β, E-cadherin, and Wnt/β-catenin signaling pathway, etc. (84). TGF-β is a crucial member of the TGF-β superfamily that is involved in EMT to regulate cell growth and differentiation (85). TGF-β1 was validated as a putative target of miR-26a-5p, which is bioinformatically analyzed to be sponged by circ_0051079 (60). The Wnt/β-catenin signaling pathway is one of the predisposing factors of EMT, which directs cancer cell migration, adhesion, invasion, and metastasis and is closely bound up with degradation of the extracellular matrix and tumor angiogenesis. This has been widely confirmed in many studies (38, 51, 72).

The ability of circRNA to affect tumor angiogenesis is usually directly related to tumor metastasis based on vascular endothelial growth factor (VEGF). Research suggests that circ_001621 augments the progression of OSA cells by abolishing the inhibition of VEGF by miR-578. The VEGF–CDK4–MMP9 axis was extended to be a novel VEGF-related pathway, which remained to be completely elucidated (31). Functional analysis by Liu et al. (66) found that circRNA_103801 as an miRNA sponge was involved in VEGF to promote tumor angiogenesis and tumor metastasis. Anoikis apoptosis is programmed cell death caused by the loss of contact between the extracellular matrix and other cells (86). Anoikis resistance was identified as a factor facilitating the progression of OSA, as metastatic OSA cells are able to colonize and survive in other sites (87). CircRNA has been found to play a role in anoikis resistance, involving Wnt pathway regulation (88). In addition, Bcl-2 and its related proteins as well as EMT processes are also involved in regulating anoikis (89). These findings are in line with studies on circUBAP2 and circ_0007534 (57, 65). Moreover, circ_0001785 showed a marked downregulation effect on antiapoptotic genes in Bcl-2 family (Bcl-W, Bcl-A1, and Bcl-2) and conversely upregulated the proapoptotic gene Bad (41).

## CircRNA and Osteosarcoma Chemotherapy Resistance

Although existing targeted chemotherapies play a role in the treatment of OSA, the emergence of drug resistance causes OSA patients to fall into an impasse. Therefore, it is important to deeply understand the mechanism of drug resistance and open new therapeutic theories. It is evident that some circRNAs participate in regulating the mechanisms of drug resistance in OSA cells. In some studies, whole-transcriptome sequencing (RNA sequencing) and next-generation sequencing technologies were performed in paired multidrug chemoresistant and chemosensitive OSA samples. Circ_0001258 inhibited the doxorubicin (DXR) resistance of OSA cell lines through upregulation of glutathione S-transferase mu 2 (GSTM2) expression *via* sponging hsa-miR-744-3p (71). Overexpression of circ_0004674 has also been observed in chemoresistant OSA cell lines and OSA patients and is negatively correlated with prognosis (28). Furthermore, Hu et al. (70) suggested that circLARP4 might elevate chemosensitivity to cisplatin and DXR *via* sponging miR-424 in OSA, closely correlated with decreased Enneking stage, better histological response, and prolonged survival profiles. Kun-Peng et al. (61) reported that circPVT1 resensitizes OSA cells to the chemotherapy drugs DXR and cisplatin by reducing the expression of the classical multidrug resistance-related gene, ABCB1. CircPVT1 could also function as a sponge for miR-205-5p to promote c-FLIP expression, thereby enhancing EMT and inducing OSA invasion and metastasis (61, 62). Hsa_circ_001569 has also been shown to enhance cell resistance to cisplatin in OSA by activating Wnt/β-catenin signaling (51). Researchers found that hsa_circ_0081001 varied in paired chemosensitive and chemoresistant OSA cell lines. It was identified as significantly upregulated in OSA cell lines, tissues, and serums and was related to poor prognosis of OSA patients. The ROC curve analysis showed that it could be used as a promising biomarker and may be a better prognostic indicator than alkaline phosphatase (ALP) and lactate dehydrogenase (LDH) (54).

## CircRNA and Osteosarcoma Prognosis

To determine the relationship between circRNA expression and prognosis in OSA, correlation analysis has shown that the expression of circRNA in OSA was related to certain prognostic factors, such as Enneking stage, tumor size, and the occurrence of distant metastasis. Meanwhile, the relationship between the expression of circRNAs and survival rates, including overall survival (OS) time and disease-free survival (DFS) time, was detected by Kaplan–Meier (KM) analysis.

Involvement of circRNAs in cancer progression has influenced the prognosis of OSA in many studies. Clinical pathologic characteristics of OSA patients and related literature were analyzed retrospectively. Xiao-Long et al. (75) found that circHIPK3 was consistently downregulated in OSA cell lines, tissues, and plasmas compared with control. Lung metastasis and advanced cancer or poor prognosis were negatively associated with lower expression levels of circHIPK3. CircHIPK3 may be used as a novel indicator for OSA with high degrees of sensitivity, specificity, and accuracy based on ROC curve analyses (73). Hsa_circ0021347 also showed the same prognostic trend (75). In contrast, research performed by Pan et al. (34) suggested that overexpression of circMMP9 was correlated with advanced tumor stage and predicted a low survival rate by KM analysis. Circ_0102049 was remarkably correlated with patients' poor OS analyzed by KM curves in a study of 76 OSA patients (35). Two studies jointly reported the role of circ-NT5C2 in OSA from the aspect of clinical and molecular biological mechanisms. Patients with a high expression of circ-NT5C2 had a shorter OS (*p* = 0.006) and DFS (*p* = 0.001) than those with a low expression of circ-NT5C2. High circ-NT5C2 expression was thought to be an independent prognostic parameter to predict poor prognosis by sponging miR-448 (52, 53). Additional upregulated circRNAs included circUBAP2 (65), hsa_circ_0000885 (45), hsa_circ_0081001 (54), hsa_circ_0004674 (28), hsa_circ_0000006 (31), hsa_circ_0051079 (60), hsa_circ_0007534 (57), and PVT1 (61).

## Osteosarcoma-Related CircRNA in Other Cancers

With advancement of circRNA research, expression of different circRNAs was detected in normal and cancerous tissue. It is worth noting that some circRNAs show similarities and participate simultaneously in different tumor types, which is similar to the basic idea of pan-cancer (90). These circRNAs are of guiding significance on the progression of multiple cancer types.

Li et al. (30) showed that the expression of circ-ITCH in OSA cancer cell lines was significantly upregulated compared with hFOB1.19. Further mechanistic studies revealed that circ-ITCH could promote the growth, migration, and invasion of OSA cells and even enhance epidermal growth factor receptor expression by reducing levels of miR-7 (30). However, another study carried out by Ren et al. (74) stated the opposite result—that circ-ITCH had lower expression in OSA cells and was identified in clinical human OSA and para-tumor tissues. Their data showed that overexpression of circ-ITCH led to reduced SP-1 expression *via* PTEN/phosphoinositide 3-kinase (PI3K)/AKT pathways, which in turn suppressed proliferation, migration, and invasion by downregulating miR-22 (74). This is in concert with the conclusion that circ-ITCH might serve as an anti-oncogene *via* sponging multiple oncogenic miRNAs in multiple tumors, including ovarian cancer, prostate cancer, melanoma, gastric cancer, glioma, breast cancer, bladder cancer, papillary thyroid cancer, lung cancer, hepatocellular carcinoma, esophageal squamous cell carcinoma, and colorectal cancer (91, 92).

Hsa_circ-0000285 has also been acknowledged to be abundantly expressed in human cells and relevant to human multicancer progression (93, 94). Hsa_circ-0000285 was believed to regulate OSA by affecting the miRNA-599/TGFB2-axis (32). It was established that hsa_circ_0000285 might act as an oncogene in laryngocarcinoma. CircPVT1 seemed to be a potential candidate of oncogenic interest. Per available literature, so far, circPVT1 has been studied to induce malignancy of different tumors including non-small-cell lung carcinoma, gastric cancer, and acute lymphoblastic leukemia (95). Additionally, many other circRNAs have been shown to participate in a variety of tumors, as follows: hsa_circ_0000190, hsa_circ_0000285, circMMP9, circEPSTI1, circ-LARP4, hsa_circ_0001785, hsa_circ_100876, circTADA2A, hsa_circ_0000502, circFAT1, CDR1as, hsa_circ_001569, hsa_circ_0003998, hsa_circ_0007534, hsa_circ_0009910, and circUBAP2. These OSA-associated circRNAs exhibit tumor-regulatory characteristics and are expected to be biomarkers in the future.

## Summary and Future Gazing

**Figure 2:**
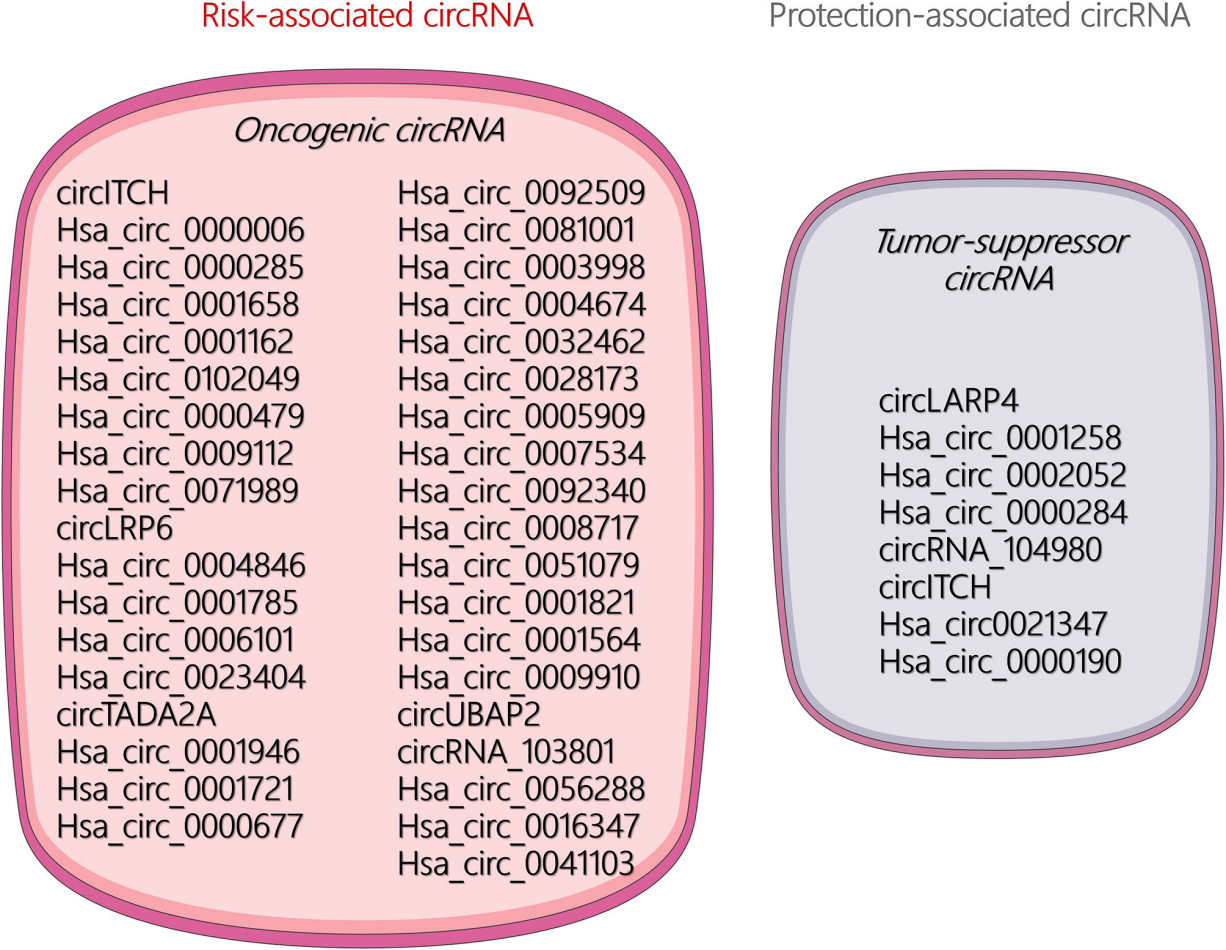
Visualization of risk- and protection-associated circular RNAs (circRNAs).

The unique circular structure of circRNA offers distinct functions and better stability than miRNA and lncRNA. Increasing research has found that circRNAs play a significant role in the occurrence, malignant progression, and metastasis in many tumors. Current research on circRNA in OSA has mainly focused on its role as an endogenous competitive RNA that acts as a molecular sponge to absorb miRNAs and thus affects the transcription of target genes. Available literature demonstrated that certain circRNAs can be regarded as risk- and protection-associated circRNAs for diagnosis of OSA characteristics, which are illustrated in Figure 2. In addition, some other biological mechanisms of circRNAs remain under investigation. For example, circRNAs may play multiple roles in the tumor microenvironment or act as noninvasive biomarkers for the early detection of cancers.

It is difficult to distinguish the source of the translation product of circRNAs because of protein-encoded exons overlapping. High-throughput analytical and detection methods such as ribosome profiling have technical challenges (96, 97). Researchers are also confronted with numerous challenges, such as difficulty in obtaining substantial tumor specimens because of the complex genetic background, extensive heterogeneity between or in tumor tissues, as well as the low morbidity. More efforts should be invested in the comprehensive evaluation of the mechanisms of circRNAs in OSA pathogenesis and chemoresistance in order to make them available for specific diagnosis and targeted gene therapy. With the research going on, we are convinced that circulating circRNAs might be used as canonical biomarkers for cancers in the future.

## Author Contributions

JL, SL, and QF reviewed relevant literature and drafted the manuscript. JL, LY, and SL conducted all data collection. All authors contributed to the article and approved the submitted version.

## Conflict of Interest

The authors declare that the research was conducted in the absence of any commercial or financial relationships that could be construed as a potential conflict of interest.
